# Towards validation of combined-accelerated stress testing through failure analysis of polyamide-based photovoltaic backsheets

**DOI:** 10.1038/s41598-021-81381-7

**Published:** 2021-01-21

**Authors:** Michael Owen-Bellini, Stephanie L. Moffitt, Archana Sinha, Ashley M. Maes, Joseph J. Meert, Todd Karin, Chris Takacs, Donald R. Jenket, James Y. Hartley, David C. Miller, Peter Hacke, Laura T. Schelhas

**Affiliations:** 1 National Renewable Energy Laboratory, Golden, CO USA; 2 SLAC National Accelerator Laboratory, Menlo Park, CA USA; 3 Sandia National Laboratories, Albuquerque, NM USA; 4 Lawrence Berkeley National Laboratory, Berkeley, CA USA

**Keywords:** Chemistry, Energy science and technology, Materials science, Mathematics and computing

## Abstract

Novel methods for advancing reliability testing of photovoltaic (PV) modules and materials have recently been developed. Combined-accelerated stress testing (C-AST) is one such method which has demonstrated reliable reproduction of some field-failures which were not reproducible by standard certification tests. To increase confidence and assist in the development of C-AST, and other new testing protocols, it is important to validate that the failure modes observed and mechanisms induced are representative of those observed in the field, and not the product of unrealistic stress conditions. Here we outline a method using appropriate materials characterization and modelling to validate the failure mechanisms induced in C-AST such that we can increase confidence in the test protocol. The method is demonstrated by applying it to a known cracking failure of a specific polyamide (PA)-based backsheet material. We found that the failure of the PA-based backsheet was a result of a combination of stress factors. Photo-oxidation from ultra-violet (UV) radiation exposure caused a reduction in fracture toughness, which ultimately lead to the cracking failure. We show that the chemical and structural changes observed in the backsheet following C-AST aging were also observed in field-aged samples. These results increase confidence that the conditions applied in C-AST are representative of the field and demonstrates our approach to validating the failure mechanisms induced.

## Introduction

During deployment, photovoltaic (PV) modules are exposed to a suite of stressors that can lead to a number of degradation modes including; encapsulant discoloration, delamination, hot spots, cell cracking, solder fatigue, potential-induced degradation, glass fracture and backsheet failure^1,2^. Aging from ultraviolet light exposure, moisture, and temperature can cause chemical changes such as polymer crosslinking or chain scission^3,4^. Additionally, mechanical stressors such as thermal expansion and wind loads can lead to cell cracking and solder or metallization fatigue^5–7^. Determining PV module design robustness against these stressors for their projected lifetimes requires validated accelerated testing methods that can reliably reproduce real-world conditions. PV module backsheets are a crucial component in conventional silicon PV modules. Their primary function is to insulate the electrical circuitry from exposure to external stressors and minimize safety risks^8^. Backsheet failure occurs when the insulating barrier is compromised, often upon the formation of cracks^9^. Failure of the backsheet poses serious safety hazards through arcing or electrical shocks^3,10^. In some cases, cracked backsheets have also led to increased leakage currents and inverter tripping, resulting in energy yield losses. Backsheet failure can also lead to an acceleration of secondary degradation mechanisms such as corrosion due to increased moisture permeation through cracks in the polymer^10,11^.

Polyamide (PA)-based backsheets gained popularity in the last decade as a less-expensive and more-recyclable alternative to historically prominent materials such as polyvinylidene fluoride (PVDF) and polyvinyl fluoride (PVF) based backsheets^12,13^. One of the first PA-based backsheets to reach commercialization was a co-extruded, three-layer composite known as “AAA”. Unfortunately, widespread failure (cracking) of AAA backsheets has been reported following as little as 4 years of field-exposure, with > 90% failure rate within 7 years of deployment^14^. The failure manifests as cracking through the entire backsheet film which presents a significant safety hazard. This typically leads to large-scale warranty claims and total module replacement efforts, incurring a significant financial cost, made more difficult by the bankruptcy of the backsheet manufacturer. With upwards of 12 GW of total deployed AAA capacity, the failure of the material is an unmitigated disaster.

Conventional accelerated stress testing (AST) protocols, such as those specified in the IEC standard 61,215-series^15^, failed to reproduce the cracking failure of AAA observed in the field. Recently, more advanced accelerated-stress testing protocols, such as the combined-accelerated stress test (C-AST)^16^ or the module-accelerated stress test (MAST)^17^ have demonstrated the ability to reproduce the observed field-failure of AAA. C-AST and MAST employ combined and/or sequential stress protocols to better show PV module weaknesses. While these new testing methods have been able to reproduce observed field failure *modes* (e.g. cracking), it is important that the enabling *mechanisms* (chemical, mechanical and structural changes) are similar to those which develop in the field. When not only failure modes but failure mechanisms can be reproduced, there is greater confidence in the testing methods and conviction that the test-induced failure is not the result of unrealistic stress application. For the C-AST method, this is of particular importance as the experimental design is based on the philosophy that no stress levels are applied that could be deemed unrealistic or unobservable in the natural environment^16^.

The development of C-AST has opened a new path forward for reliability testing of PV modules and materials, but further validation of the degradation mechanisms induced is an important step to justifying widespread application. In this work, we apply various techniques to characterize the chemical and structural characteristics of unaged, field-aged, and C-AST-aged AAA backsheet samples. These techniques include optical microscopy, Fourier-transform infrared spectroscopy (FTIR), wide-angle X-ray scattering (WAXS), X-ray transmission imaging, and differential scanning calorimetry (DSC), along with Finite Element Modelling (FEM) of respective stress environments. Through this characterization, we compare changes in material properties from the field to C-AST and determine that the mechanisms induced by C-AST are representative. The results of this work provide support for C-AST as a valid accelerated stress testing protocol and outlines an approach to validating novel stress testing protocols which goes beyond just demonstrating reproducibility of degradation *modes.*

## Materials and methods

### Characterization techniques

Various techniques are employed to characterize the backsheet materials. These include optical microscopy, Fourier-transform infrared spectroscopy (FTIR), wide-angle X-ray scattering (WAXS), X-ray transmission imaging, and differential scanning calorimetry (DSC). Additional information on the methods may be found in the supplementary information (SI) section.

### Combined-accelerated stress testing

The C-AST capability, developed within a modified XR-260 weathering chamber (Atlas Material Testing Technology, LLC), combines multiple stress factors of the natural environment and the conditions experienced by PV modules in the field and has been previously described in detail elsewhere^18^. In summary, this approach allows for the detection of degradation mechanisms including those not known a priori in new PV module designs or bills of materials. The capability allows air temperature control, relative humidity (RH) control, full spectrum Xenon-arc lamp irradiance front-side mechanical pressure, system voltage bias and water spray^18^. The test protocol applied is a modified version of ASTM D7869^19^ (Fig. S1). A nominal front-side UV radiation of 0.8 Wm^2^/nm at 340 nm is applied, with aluminum trough reflectors mounted beneath the sample plane delivering a rear-side UV radiation of 0.12 W/m^2^ at 340 nm (approximate 5–15% reflection). Module temperature was controlled between an upper limit of 90 °C ± 5 °C and − 20 °C and relative humidity varies between 95 and 28%, including with the application of a water spray at specific intervals. Mechanical loading up to the equivalent of a 2400 Pa load on 60-cell modules is applied through the vertical displacement of ring-shaped actuators on the front surface of the test samples. The displacement determination has been previously described^16^.

### Finite-element analysis

A finite-element model of the C-AST MiMo was developed to quantify peak stresses occurring in a typical tabbed solar cell geometry under thermal and mechanical conditions^20^. Models use C-AST mini-module dimensions for features such as cell spacing, cell tab thickness, solder height, and laminate layer thicknesses (Fig. S2). The module was simplified as quarter symmetric, with all test features such as the loading ring and support frame explicitly modeled (Fig. S2). The geometry was discretized using CUBIT software^21^ with 2.7 million linear hexahedral elements distributed over the quarter symmetric domain, with conformal meshing applied to laminated interfaces and non-conformal frictional contact interfaces applied between the MiMo and test fixture. An additional 10-million element mesh was generated to verify convergence of modeled quantities of interest to within approximately 1% of nominal predicted values. Thermoelastic material properties are implemented for EVA and backsheet based on measured values along with secant coefficients of thermal expansion^22^, while linear elastic properties are used for the glass, silicon, and metallic materials Boundary conditions include a 1.1 mm fixed displacement in the negative y-direction applied to the loading ring (Fig. S2), to force the mini-module to attain a similar curvature as a full module experiencing a − 2400 Pa pressure load^20,23^ both at a 25 °C reference state and at − 20 °C and 90 °C to match C-AST Tropical cycle temperature states^16^. Simulations were run using the SIERRA/Adagio code^24^.

## Test samples

**Figure 1:**
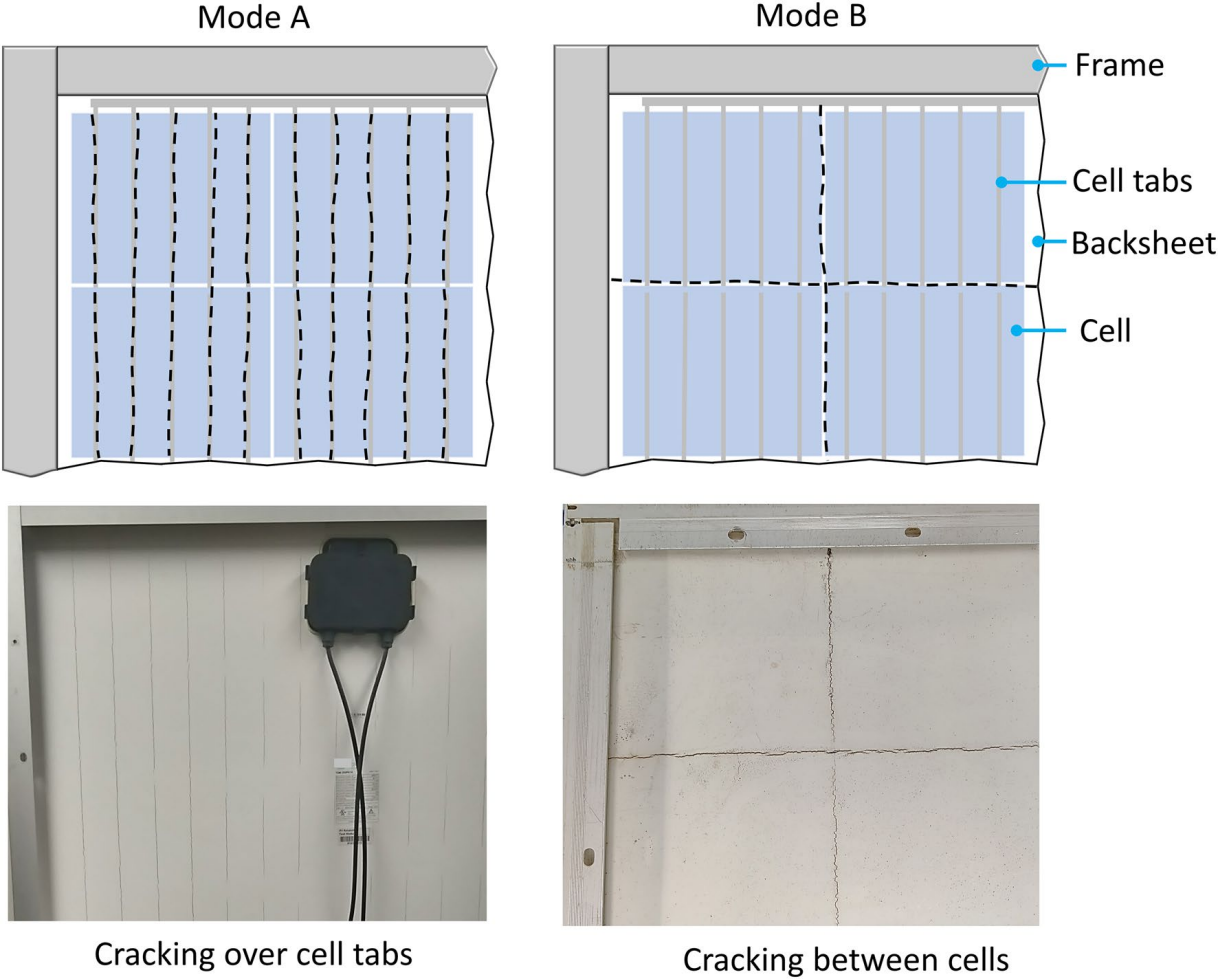
Schematic of the observed cracking modes in the backsheet with Mode A; cracking over cell ribbons and Mode B; cracking in the area between cells. Black dashed lines indicate crack location and orientation. Blue squares indicate relative cell location.

The inner and outer layers of AAA are composed of polyamide-12 (PA12) with rutile TiO_2_ white pigment measuring 38 μm ± 5 μm in thickness and a PA-12/polypropylene (PP) blended core layer with glass fiber filler measuring 285 μm in thickness. Tinuvin 234 UV absorber, Tinuvin 770 light stabilizer, Irganox 1010 ephenolic antioxidant and ADK AO 80 hinderd phenol antioxidant^25^ are also added to stabilize the material^25^. Two sample sets are included in the study. The first is made up of backsheet extractions from four fielded modules. Modules came from two different manufacturers and the exact bill-of-materials (BOM) was unknown. The fielded modules had been deployed in various locations for varying lengths of time (as summarized in Table S1). Each module has 60 mono-crystalline silicon cells, a 3.5 mm glass superstrate, a poly(ethylene-co-vinyl acetate) (EVA) encapsulant, AAA backsheet substrate, and an aluminum frame. Both macrocracking and microcracking were observed in the backsheet samples. Macrocracks are large cracks which are visible to the naked eye, with width of approximately 0.5 mm. Microcracks are not visible to the naked eye but observable under magnification, with typical widths of approximately 1–5 μm. Two cracking modes are observed in the fielded modules. The first is described as macrocracks which occurred only in the machine direction of the polyamide backsheet and conformed with underlying features i.e. the cell tabbing (Mode A, Fig. 1). The second cracking mode saw macro-cracks in both the machine and transverse directions and located within the spacing between cells (Mode B, Fig. 1). Field-aged samples were also accompanied by the well-known “chalking” phenomenon, whereby the outer surface of the backsheet is eroded and leaves a fine residue when wiped^26^.

The second sample set includes artificially aged samples (Table S2). Two miniature-modules (“MiMos”) (C-AST-1 and C-AST-2) with four crystalline silicon cells soldered in a 2 × 2 configuration are laminated together with a glass front sheet, EVA encapsulant and AAA backsheet. C-AST-1 contains a UV blocking (360 nm cut-off), while C-AST-2 contains a UV transmitting EVA. A junction box is adhered to the rear-surface to realize fully functional devices. These devices are used for testing connected to a load resistor in the C-AST protocol. C-AST-1 is subjected to 84 days in the C-AST Tropical cycle, whereas C-AST-2 is subjected to a total of 184 days in the C-AST Tropical cycle.

Three additional samples are laminated with the same glass, encapsulant, and backsheet under the same lamination conditions. These samples contained only a single cell and no junction box. The first sample (T) is exposed to an extended 90 °C temperature soak in the dark with nominal 10% RH totalling 2000 h. The second sample (UV) is exposed to a UV soak at 0.4 W/m^2^ at 340 nm at 45 °C and ambient humidity levels, totalling 1500 h. The third sample (Unaged) is used as a reference for characterization of the AAA backsheet “as-received”.

## Results and analysis

Here we present the findings from a series of characterizations that allow us to develop a complete picture of the *mechanisms* responsible for the observed degradation *modes.* In each sub-section we present the results from the respective characterization method and draw comparisons between field-aged samples and C-AST-aged samples.

### Optical and X-ray imaging

**Figure 2:**
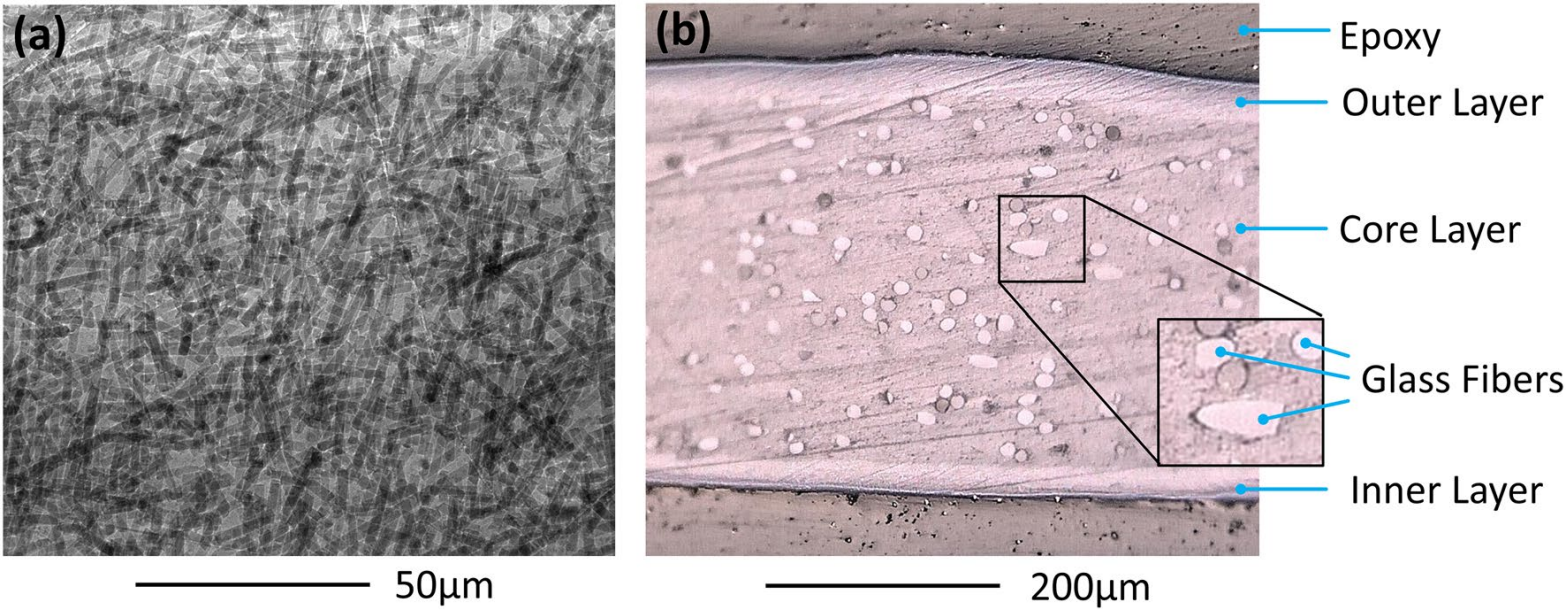
(**a**) X-ray transmission image (normal to the surface) and (**b**) a cross-sectional micrograph of the as received backsheet showing the glass fiber filler present in the core layer.

As described in Sect. 2.4, the AAA backsheet contains a glass fiber filler in the core layer. The glass fiber filler is observable using X-ray transmission imaging (as shown in Fig. 2). Given the high density of glass rods in the backsheet, we explore the role this additive may have in crack formation. The X-ray and optical cross-section imaging in Fig. 1 show that the fibers are randomly oriented in-plane. We further analyzed the in-plane rod directionality using local gradient and wavelet decomposition methods, finding possible subtle alignment effects and spatial heterogeneity. However, a full description is beyond the scope of this work. Furthermore, no cracking was seen to initiate, or concentrate, around the periphery of the glass fibers suggesting that their distribution likely had little influence on the initiation of cracking in the backsheet.

**Figure 3:**
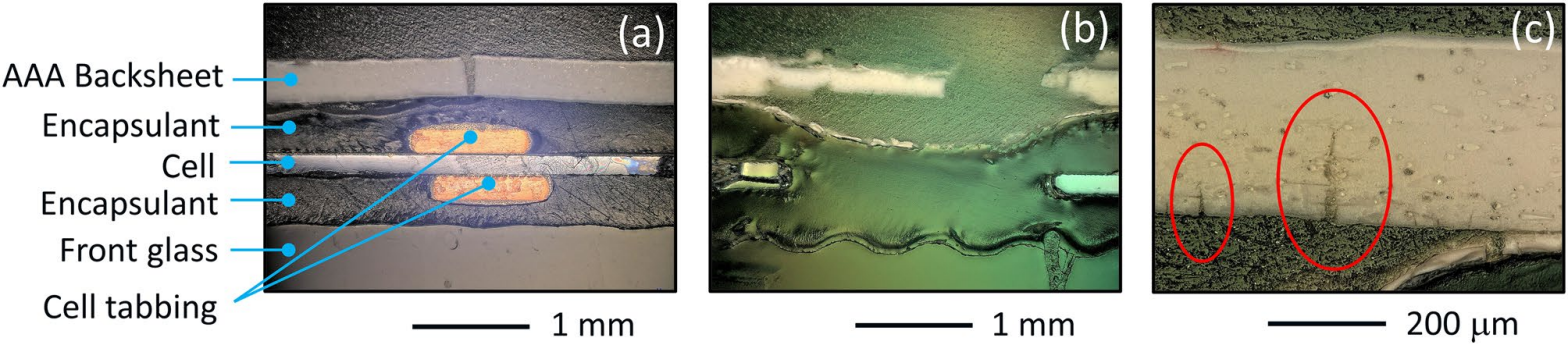
Cross-sectional micrographs of (**a**) the C-AST-2 sample featuring a macrocrack over a cell tab, (**b**) the Rome sample featuring a macrocrack at the space between two cells, and (**c**) the Rome sample featuring two macrocracks in the core layer (also at the space between two cells) which can be seen to propagate from the inner-layer outward.

Representative images of cross-sections for samples C-AST-2 and Rome are given in Fig. 3, where C-AST-2 demonstrated Mode A cracking (located over cell tabs) and Rome demonstrated Mode B cracking (occurring between cells). Figure 3a shows a micrograps of sample C-AST-2, including the region over a cell tabbing. The macrocracks in C-AST-2 coincide with the cell tabbing, where the crack is located directly over the cell tabs, as in Mode A cracking. The convex shape of the backsheet over the ribbon is expected to give localized tensile stress (facilitating crack formation and propagation) at the outside (air-facing) surface of the backsheet. In addition to the macrocrack in the C-AST-2 sample (Fig. 3a) the outer surface of the backsheet sample demonstrates a network of microcracks (verified on the sample at greater magnification), suggesting that a change in mechanical characteristics (i.e., fracture toughness possibly due to chemical degradation as well as biaxial shrinkage) is occurring in that layer. Microcracks are not observed in the inner (cell side) layer. The combination of tensile stress and change in mechanical characteristics is believed to result in macrocrack development, with the crack propagating from the outer layer to the inner layer. In addition to C-AST, Mode A cracks have also been observed in fielded modules^27^.

Figure 3b shows a micrograph of the Rome sample including the region between two cells. In this case, Mode B macrocracks are seen to develop within the spacing between the cells. The local thickness of the encapsulant is reduced between the cells because no cell is present at that location and the encapsulant is able to locally flow during lamination. The concave shape of the inner backsheet layer between the cells would be expected to give a localized tensile stress at the inside (cell-side) surface of the backsheet. Figure 3c shows a micrograph from another location of the Rome sample, also taken in the spacing between two cells. In this case, propagating cracks are observed at the cell-side of the core layer of the backsheet (circled in red). Closer examination of the Rome sample (Fig. 3b,c) reveals that the inner layer of the backsheet has numerous smaller microcracks and is also locally delaminated from the rest of the backsheet in the region between cells. Between cells, UV light can reach the backsheet material through the cell-side of the module. Microcracking and delamination suggest a change in mechanical characteristics of the backsheet; the locations of those features suggest degradation is facilitated by photo-degradation. Microcracking and delamination were not observed on the cell-side of the backsheet in regions masked by PV cells. As in the C-AST-2 sample (Fig. 3a, the macrocracking observed in the Rome sample (Fig. 3b,c) is believed to result from the combination of tensile stress and change in mechanical characteristics. The location of the cracks in the Rome sample (Fig. 3c) is consistent with crack initiation on the cell side of the backsheet, with subsequent propagation occurring through the core in the direction of the air side. Once formed, subsequent crack propagation may result from misfit stress from the mechanical- or thermomechanical-history of the module^28^.

Overall, we observe that the local topography (underlying cell tabbing or recess between cells) may facilitate the development of macrocracks, which are likely initiated from microcracking of the inner or outer layers. With sufficient aging, damage can continue at the local topography site (including the formation of a network of cracks and/or delamination), resulting in loss of a region of the backsheet from the module as in Fig. 3. The propagation of macrocracking due to local topography is a consistent observation in both C-AST-aged samples and field-aged samples.

### Finite-element analysis of C-AST mechanical stressors and locations

**Figure 4:**
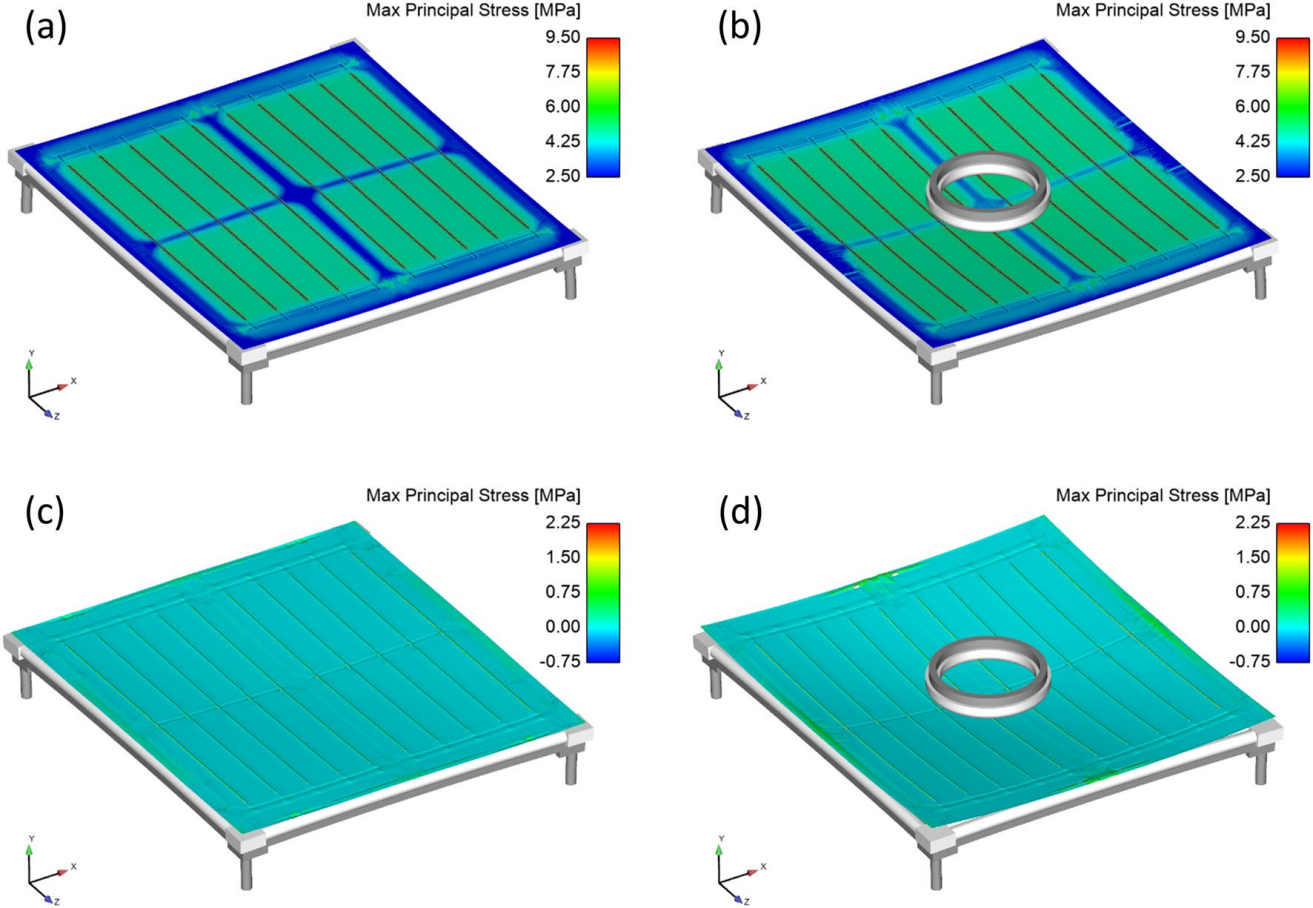
Simulated principal stress on backsheet for C-AST MiMos at (**a**) − 20 °C without mechanical load, (**b**) − 20 °C with mechanical load, (**c**) 90 °C without mechanical load and (**d**) 90 °C with mechanical load showing stress concentrations over cell tabbing lengths but minimal change due to mechanical loading. Displacements scaled by 10 ×. Graphics rendered using Sierra/SolidMechanics 4.56, https://www.sandia.gov/ASC/integrated_codes.html.

Simulation results of the C-AST MiMos were examined to identify the environmental situations promoting macrocrack formation. Results showed that mechanical loading alone produced concentrations in backsheet stress over cell gap areas as observed in Mode B cracking (Fig. S3). However, the magnitude of these stresses was significantly lower than thermally induced stresses due to encapsulant and backsheet contraction and stiffening, particularly at − 20 °C where the largest stresses overwhelmingly occurred in Mode A over rigid interconnect topology, regardless of whether mechanical load was applied (Fig. 4a,b). At 90 °C, stresses throughout the MiMo were reduced due to decreasing material elastic modulus with increasing temperature, which also limited the apparent impact of mechanical load (Fig. 4c,d). Key insights from the finite element model were that low temperature excursions (− 20 °C) leading to Mode A cracking were the most likely failure mode to be expected for C-AST, consistent with the Mode A cracking observed in the C-AST-2 sample. In addition, although they were of a lower magnitude than thermal stresses, Mode B cracking stresses were still activated during mechanical loading at moderate temperatures, consistent with the sampled field environments of the Rome and Bergamo modules where Mode B cracking was observed. Only extreme high temperatures of C-AST were found to be minimally influential to crack activation. C-AST conditions can thus be considered a conservative replication of the thermal mechanical stressors of field environments, since they enter conservatively low temperature regimes favouring the observed Mode A cracking, but nonetheless successfully activate the same Mode B cracking stresses observed in field samples.

### Chemical analysis

**Figure 5:**
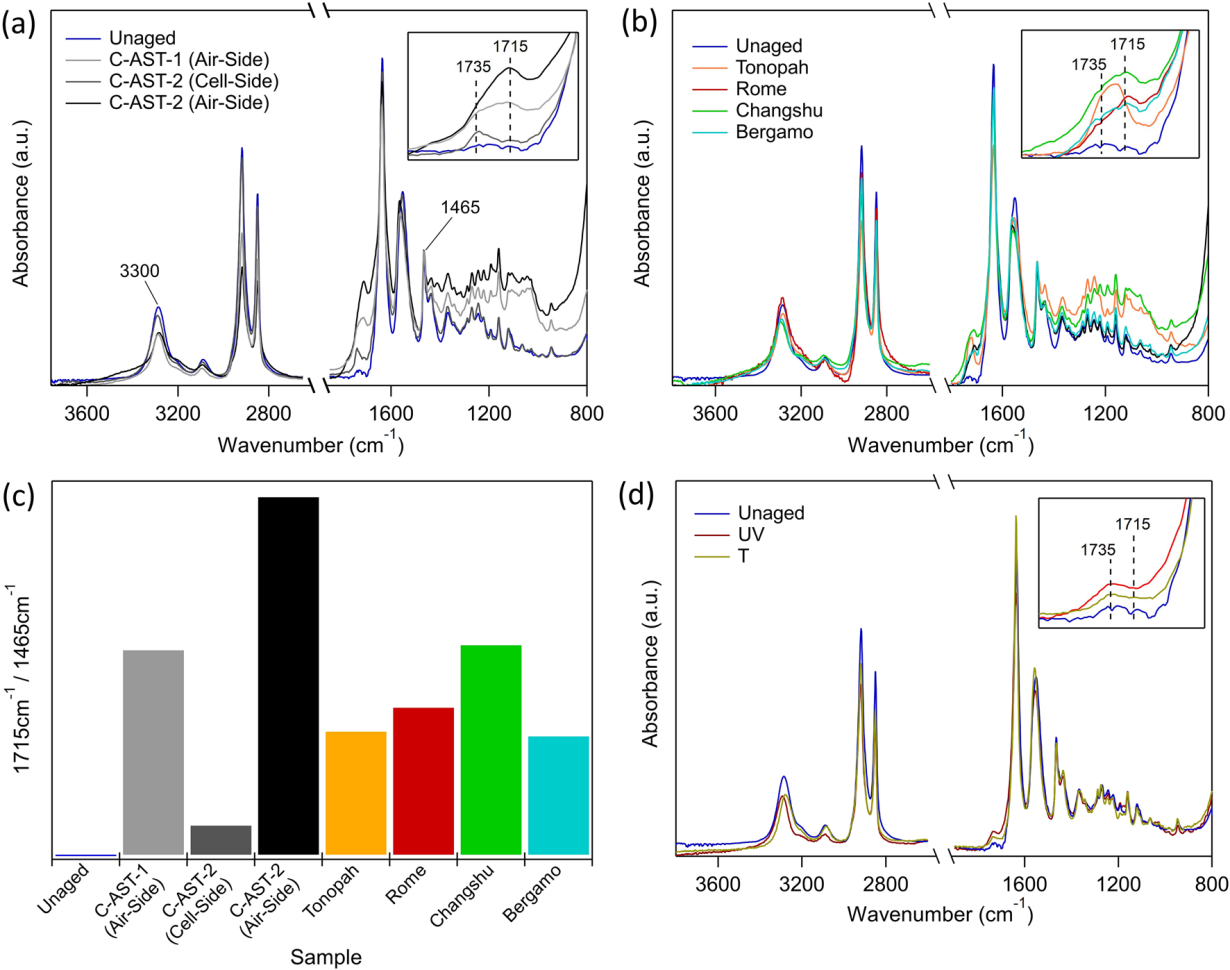
FTIR-ATR spectra for (**a**) an unaged and C-AST aged AAA samples, (**b**) field-aged AAA samples measured from the air-side, (**c**) air-side measurements from unaged, UV and T AAA samples and (**d**) relative changes in 1715 cm^-1^ peak height for all samples.

FTIR-ATR is employed to inform any chemical changes occurring in the AAA backsheet samples during aging. Chemical changes in polymers are often associated with a change in mechanical properties. Two primary observations are made. First, the broadening of the shoulders around the 3300 cm^−1^ peak from the air-side of the C-AST-2 sample, suggesting the formation of hydroxylated products and/or primary amines, such as carboxylic acids^29^. The second observation is the increasing intensity of the peak in the range of 1700–1740 cm^−1^. On closer inspection there are two distinct peaks present here (Fig. 5a, inset). The first peak which centers at 1715 cm^−1^, and a second peak which centers at 1735 cm^−1^. The 1735 cm^−1^ peak is associated with imide groups which can be the product of photo-degradation reactions^31^. Imides are known to be unstable and convert to carboxylic acids in the presence of water^32^. The peak at 1715 cm^−1^ suggests carbonyl group products, namely carboxylic acid, and could be explained by moisture-induced breakdown of imide groups^30^. The photo-oxidation peaks 1715–1740 cm^−1^ are only observed for the air-side layers of AAA, which were exposed to UV. The same peaks are not observed in the cell-side layers, which are not exposed to UV light. In previous studies, the photo-oxidation process has been attributed to a breakdown of the amorphous regions of PA-12 which is then followed by chemi-crystallization leading to a change in mechanical characteristics (i.e. a reduction in fracture toughness)^30^. The change in crystallinity is characterized using WAXS and DSC and discussed further in the following sections of this paper.

Figure 5b presents the collected spectra for the outer layers of the fielded AAA samples. Similar increases in the 1700–1740 cm^−1^ peak intensities are observed in each of the fielded samples as compared to the C-AST-aged samples. The consistent peak increase in C-AST- and field-aged samples suggests that a similar photo-oxidation mechanism is happening on the outer surface (exposed to UV), which leads to the observed microcracking. However, there are some differences in the intensity of the 1715 cm^−1^ and 1735 cm^−1^ between the fielded samples, where the Tonopah sample demonstrated a more pronounced peak at 1735 cm^−1^ (the imide groups), while the other samples demonstrated more pronounced 1715 cm^−1^ peaks (carboxylic acids). The lower humidity and moisture conditions of Tonopah, AZ could explain the more pronounced imide group peak, where the lack of moisture means the imide groups do not break down into carboxylic acid as in the other samples, which experience more humid environments. Previous work has demonstrated that imide groups are favoured at the inner layer of fielded modules, except in the case where macrocracking is present and moisture can more easily penetrate the backsheet^33^. In the case of the C-AST-aged samples, we see that C-AST-1 (which exhibited no macrocracking) demonstrated a higher imide-to-carboxylic acid ratio than C-AST-2 (which exhibited extensive macrocracking). Similar peak intensity increases have also been widely reported for field-aged and some laboratory aged AAA samples^27,33,34^.

Figure 5c shows the change in normalized peak intensity for the carbonyl peak 1715 cm^−1^ for the C-AST- and field-aged samples from the outer layer of the backsheet. Previous studies used the height ratio of the carbonyl peak at 1715 cm^−1^ to the normalization peak at 1465 cm^−1^ as a measure of polymer decomposition^33^. The peak intensity could be interpreted as the degree of UV photodegradation. We show that C-AST-2 has the highest change in intensity on the outer layer, however, it is worth noting that C-AST-2 was aged in C-AST for an additional 3 weeks after the initial formation of macrocracks. Of the fielded samples, Changshu had the greatest change in peak intensity, followed by Rome, Tonopah and Bergamo. Rome and Bergamo were the only samples to demonstrate Mode B macrocracking, which was initiated from the inner layer. Additionally, these samples demonstrated minimal outer layer microcracking under magnification. The severity of the cracking of the Rome and Bergamo samples would suggest that some other chemical degradation is occurring which allows for macrocrack formation at the inner layer of the backsheet. Acetic acid is a known by-product in EVA resulting from the hydrolysis of the vinyl-acetate monomers^35^. A recent study demonstrated that the chemical degradation of free-standing AAA exposed to the outdoors could be accelerated in the presence of acetic acid^33^.

To further understand the observed mechanisms, and decouple the effects of UV and temperature, an additional backsheet two samples were subjected to a dark temperature soak (90 °C, 5% *RH*) and UV soak (0.4 W/m^2^ at 340 nm, 45 °C) for 2000 h and 1500 h, respectively. FTIR-ATR spectra were collected from the air-side layers of each of these samples, as shown in Fig. 5d. Neither the T nor UV sample demonstrates strong changes in peak intensity comparable to those observed for the samples aged in C-AST or the field. However, the UV sample does show a slight emergence of the 1735 cm^−1^ peak to suggest that some amount of photo-oxidation is occurring. The total UV dose applied to the UV sample was 2.16 MJ/m^2^ at 340 nm. By comparison, C-AST-1 and C-AST-2 samples were exposed to 0.25 MJ/m^2^ and 0.5 MJ/m^2^ at 340 nm, respectively. Chalking was also observed for the T sample, but there was no evidence of substantial chemical degradation in the FTIR spectra. The T sample was exposed to 2000 h at 90 °C. In C-AST, samples C-AST-1 and C-AST-2 were exposed to a total of 720 h and 1440 h at 90 °C, respectively. This suggests that while photo-oxidation can occur with exposure to UV light, the reaction appears to be thermally activated such that UV exposure at lower temperatures (as was performed on the UV sample) does not facilitate the same degree of chemical degradation as C-AST. Most crucially, the FTIR measurements imply that C-AST causes chemical decomposition of AAA backsheets to a similar magnitude as does field aging. In addition, the small changes in the FTIR spectra that were present for samples T and UV favoured the 1735 cm^−1^ peak, to suggest the formation of imides, and not carboxylic acids. Neither UV or T samples were exposed to elevated moisture or relative humidity conditions, which further supports the humidity requirements for formation of carboxylic acids from imide groups, as seen in both the field and in C-AST.

### Analysis of polymer crystallinity

**Figure 6:**
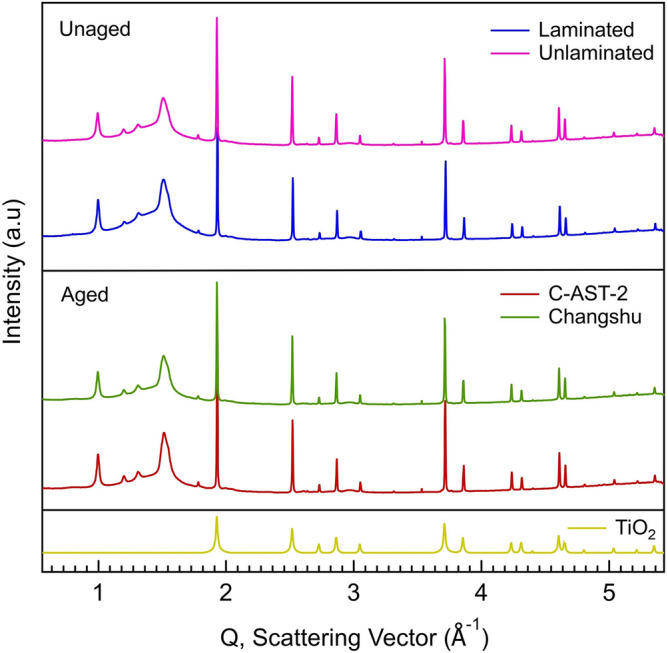
WAXS of unaged and aged AAA backsheets. X-ray scattering of unlaminated (magenta), laminated (blue), C-AST-2 (red), and a representative field aged sample, Changshu (green). The reference pattern for the rutile phase of TiO_2_ is included (gold). Q is related to the diffraction angle (θ) and incident wavelength (λ) by Q = (4π/λ) sinθ. The X-ray wavelength used here was λ = 0.9744 Å.

X-ray scattering can provide insight into the crystalline phases present in the backsheet. Here we use WAXS to determine if any structural changes are occurring in the backsheets from aging. We also examine the potential impact of lamination on the backsheet by comparing laminated and unlaminated. Many previous studies focus on the aging behaviour of unlaminated, freestanding backsheet samples. Figure 6 shows scattering data for the unaged (laminated and unlaminated), C-AST aged (C-AST-2, 184 days), and a representative field-aged (Changshu) AAA backsheet sample. The sharp and intense peaks are indexed to the rutile-TiO_2_ pigment present in the backsheet. The remaining broader peaks, mostly located in the lower Q region, below 2.0 Å^-1^, are due to scattering from the semi-crystalline polyamide polymer. There are no major changes to the scattering for any of the samples studied here, which allows us to make two major conclusions. First, the lamination process does not appear to affect the polymer or TiO_2_ structure. A major change in the structure of TiO_2_ might compromise the barrier coating on that material, enabling photodegradation of the backsheet from within by TiO_2_, rather than UV protection by optical scattering. Second, we observe no additional peaks after aging both in the field and through C-AST, suggesting the chemical changes observed in FTIR do not present as novel crystalline phases in the backsheet. The C-AST and Changshu samples show a slight increase in the intensity of the polymer peaks compared to the laminated sample suggesting a slight increase in the overall crystallinity of the backsheet. However, quantification of crystallinity by WAXS is challenging and more insight can be gained through DSC.

**Figure 7:**
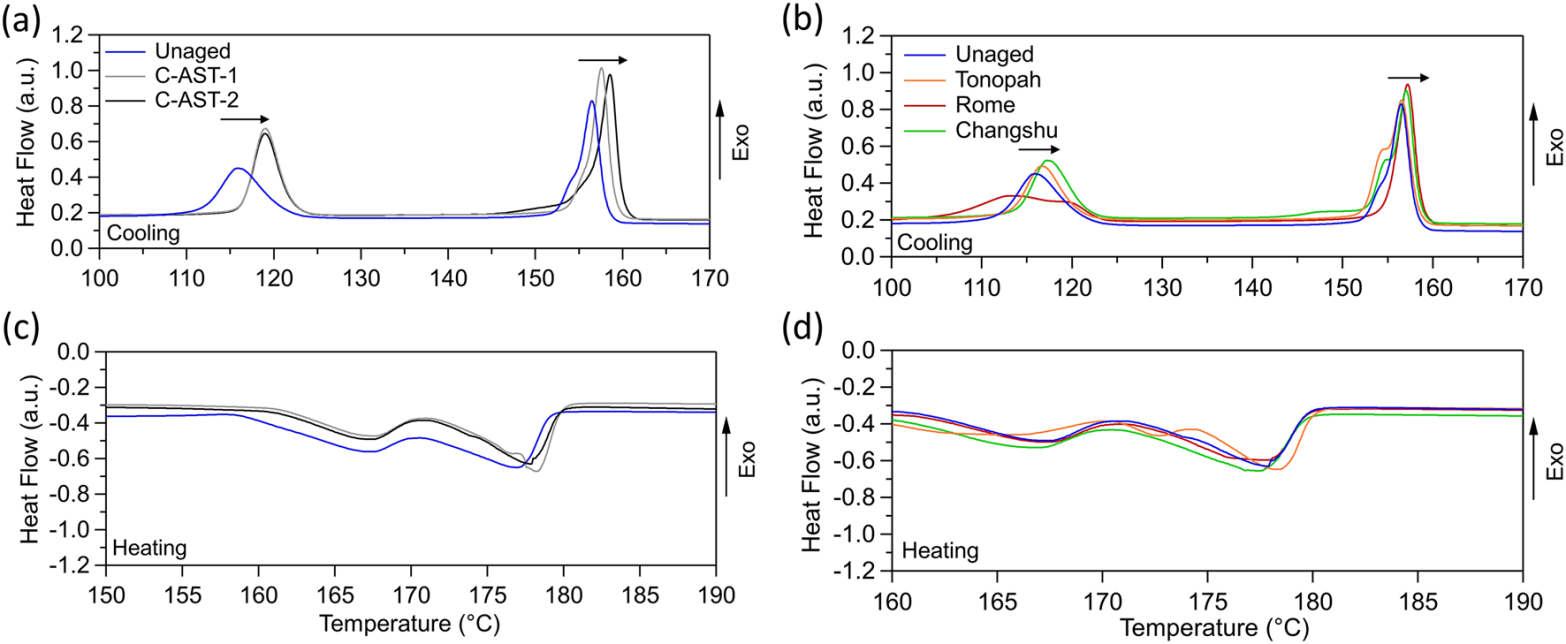
DSC heat flow for (**a**) first cooling cycle for unaged and C-AST-aged AAA samples, (**b**) first cooling cycle for unaged and field-aged samples, (**c**) first heating cycle for unaged and C-AST-aged samples and (**d**) first heating cycle for unaged and field-aged samples. Arrows indicate trends of the key thermal transition peaks.

Normalized heat flow data collected by DSC is shown in Fig. 7. Two main recrystallization thermal transitions are observed during the cooling cycle in all samples, around 115 °C and 155 °C. These are attributed to the polypropylene and polyamide-12 components, respectively^36^. In Fig. 7a it is shown that the crystallization temperature of the C-AST aged samples increased by around 5 °C (as compared to the unaged sample) for the polypropylene component, and around 3 °C for the polyamide-12 component. A similar trend is observed for the field-aged samples shown in Fig. 7b (except for Rome). The cooling curve for Rome in Fig. 7b shows a different polypropylene transition, instead showing a double peak at 111 °C and 120 °C, and also lacks the peak at 153 °C in the polyamide-12 transition demonstrated by the other samples. Interestingly, Rome was the only sample in the set which showed Mode B cracking (cracking driven from the inner side between cells).

DSC data from the first heating cycle (Fig. 7c,d), show two endothermic peaks in all samples around 165 °C and 180 °C. These peaks are associated with the melting of two crystalline phases (polypropylene and polyamide-12, respectively) within the backsheet polymer network.

**Figure 8:**
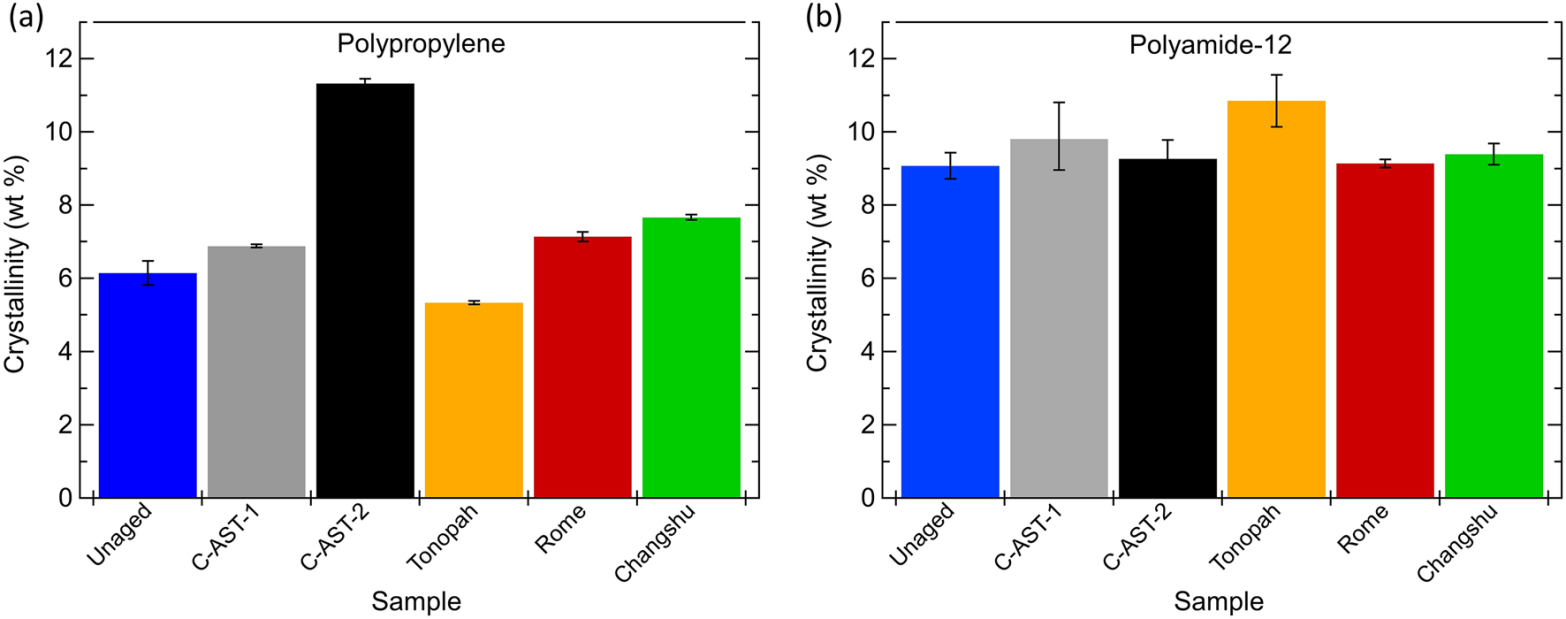
Crystal weight (%) for (**a**) the polypropylene component and (**b**) polyamide-12 component of the AAA backsheet samples. Error bars represent 2 standard deviations.

Each melting peak can be integrated to calculate crystal weight % using (1). Calculated polypropylene and polyamide-12 crystal fractions for each sample are included in Fig. 8a and b, respectively. Since this calculation was completed with heat flow data from the first heating cycle, the results are representative of the polymer state “as is” in the module allowing us to infer the state of the polymers after aging.

1 $$Crystallinity \left(wt\%\right)=\frac{{\Delta {\rm H}m}_{measured}}{{\Delta {\rm H}m}_{ reference}}$$

where ${\Delta {\rm H}m}_{measured}$ is the integrated area in J/g, ${\Delta {\rm H}m}_{ reference}$ for polypropylene and polyamide-12 crystals are 207.0 J/g and 209.3 J/g, respectively ^37^. Only the core layer of the backsheet contains polypropylene, where polyamide-12 is present throughout the layers, with the inner and outer layers containing only polyamide-12. In Fig. 8 it is shown that the crystal weight of the polypropylene component in most samples increases slightly with aging, with Tonopah being the exception. The most significant increase is observed in the C-AST-2 sample. No significant change in crystallinity is observed in for the polyamide-12 component. Of the field-aged samples, Changshu is shown to have the highest crystalline content for the polypropylene component, higher than Rome. However, Changshu only exhibited outer layer micro-cracking, and not macrocracking. Whereas Rome exhibited significant macro-cracking in Mode B (from the inner layer), and less outer layer microcracking. The C-AST-2 sample demonstrated significant macro-cracking in Mode A (over the cell tabs), as well as the most significant microcracking of the outer layer and degree of outer layer photo-oxidation as measured by FTIR (Fig. 5c). The DSC results presented does not allow for a correlation between change in crystallinity and occurrence of either microcracking of the outer layer, or macrocracking through the entire backsheet film.

Previous work has demonstrated a change in the crystallization properties of polypropylene in the AAA backsheet following damp heat aging (85 °C/85% *RH*), followed by a reduction in elongation-to-break properties^27^. The change in crystallinity observed for each of the fielded samples in Fig. 8a demonstrates a correlation with climate classification, where Changshu is classified as humid sub-tropical, Rome is humid continental and Tonopah is cold desert, according to the Köppen-Geiger climate classification system^38^. As such, Changshu and Rome would have been exposed to high humid and high temperature environments, with Changshu presenting the more severe conditions. Whereas Tonopah would have been exposed to low humidity conditions. This is also supported by the presence of the imide to carboxyl group ratios from the FTIR data (Fig. 5b), where Tonopah saw a higher ratio of imide groups than the other samples since there would have been insufficient moisture to convert the imides to carboxylic acids^31^. A similar correlation was observed in the elastic modulus of individual layers of AAA backsheet from modules fielded in the same location, where in this case the elastic modulus was used as a proxy for fracture toughness^33^.

While Changshu demonstrated the greatest increase in crystallinity of the propylene content, it did not suffer macrocracking in the same way as Rome, which did not increase as much. However, it is again worth noting that Rome exhibited Mode B cracking (inner layer, between cells), which was likely driven by another mechanism such as acetic acid attack. The C-AST-2 sample demonstrated the greatest increase in polypropylene crystallinity but was the only sample in this study to demonstrate Mode A cracking (over cell tabs). The C-AST-1 sample also demonstrated an increased polypropylene crystallinity approximately equivalent to Rome, but less than Changshu. The C-AST-1 sample did not exhibit any macrocracking.

The results here show that crystallization of the polypropylene in the core layer of the AAA backsheet is occurring. This crystallization could be responsible for a loss of mechanical characteristics which allow macrocracks to propagate throughout the entire backsheet stack. Furthermore, it would appear as though a greater degree of crystallization is required to induce Mode A cracking (over cells tabs) than Mode B cracking (between cell gaps).

## Discussion

The results presented in this work point to multiple, complex degradation mechanisms leading to two different cracking failure modes of the AAA backsheet. From microscopy and FTIR, a UV-induced photo-oxidation mechanism is attributed to the observed chemical degradation on the outer (light exposed) layers of the AAA backsheet, which is not observed in areas of the backsheet shaded from light exposure (Fig. 5). Two peaks at 1715 cm^−1^ and 1735 cm^−1^ could be observed in the FTIR spectra (Fig. 5a and b) which are associated with photo-oxidation mechanisms. The peak at 1735 cm^−1^ could be associated with imide groups, which are a product of photo-oxidation. Imide groups are unstable and susceptible to breakdown into carboxylic acids in the presence of moisture. A discrepancy was observed in the peak intensities of 1715 cm^−1^ and 1735 cm^−1^ between the samples, more specifically, the sample from Tonopah, AZ, exhibited a greater peak intensity at 1735 cm^−1^, while the other samples exhibited a great peak intensity at 1715 cm^−1^. This discrepancy could be explained by the lower *RH* in Tonopah, AZ, which limits the imide group breakdown into carboxylic acids.

Through FTIR analysis it was shown that the C-AST-2 sample exhibited the highest amount of chemical degradation and demonstrated severe cracking over the cell tabs (i.e. Mode A). Of the field-aged samples, Rome was the most damaged, having suffered significant macrocracking in the space between cells (i.e. Mode B). However, in FTIR, Rome exhibited less chemical degradation than Changshu, which saw no macro-cracking. Cross-sectional optical microscopy of the backsheets revealed that the macro-cracking mode observed in the Rome sample was likely initiating from the inner layer (Fig. 3c). Polyamide is known to be vulnerable to acids (degradation catalysed by acids), and photo-oxidation of the AAA backsheet inner and outer layers is demonstrably accelerated in the presence of acetic acid^33^. Therefore, a possible explanation for the discrepancy in cracking modes observed in field-aged samples from different locations could be due to the quality of the EVA encapsulant.

DSC suggested that an increasing crystallinity of the polypropylene content was occurring with aging in all samples but Tonopah (Fig. 8). It has been previously shown that this change in crystallinity can occur as a result of exposure to the damp heat condition (85 °C/85% *RH*), and leads to a significant reduction in elongation-to-break, to suggest that it negatively affects the mechanical characteristics of the AAA core layer^27^. Similar changes in mechanical characteristics have been observed for elevated temperature exposures with lower *RH* conditions^39^. Crystallization of polypropylene can be caused by extended exposures to elevated temperatures^40^. A correlation was identified between deployment time of the fielded samples, and the degree of crystallinity of the polypropylene, where the greatest change in crystallinity was observed for samples deployed in the field for the longest time (Rome and Changshu, 5 years and 4 years, respectively). While elongation-to-break may not be directly related to the fracture potential of the polymer, additional work has demonstrated a similar correlation between climate and elastic modulus of the backsheet, which further supports the notion that elevated humidity leads to a detrimental change in mechanical characteristics of the AAA backsheet and could be directly related to cracking potential^33^. In this study, C-AST-2 had the greatest increase in polypropylene crystallinity, which could have alluded to possible unrealistic stress conditions in C-AST. However, C-AST-2 was the only sample to demonstrate Mode A cracking (over cell tabs). The Changshu sample had the second-highest degree of crystallinity (after C-AST-2), but demonstrated no macrocracking, while the Rome sample demonstrated significant macrocracking in Mode B (between cells), with a lower crystallinity. All of this suggests that Mode A cracking (over cell tabs), requires additional aging with increased polypropylene crystallization and outer layer photo-oxidation to initiate compared to Mode B cracking (between cells). Had the Changshu sample remained in the field for longer, it would have likely developed Mode A cracking like that of the C-AST-2 sample. To validate this, analysis could be conducted on a field-aged sample which exhibits Mode A cracking.

Cracking of the AAA backsheet is seemingly a two-step process requiring chemical degradation to initiate micro-cracking, crystallization of the polypropylene core layer, and subsequent mechanical loading to propagate macrocracking through the entire backsheet. The FEM presented in this work shows that localized stress concentrations are present on the backsheet in the regions directly above the cell tabs, and in the space between cells. These stresses are driven largely by thermal cycling, where the mechanical loading conditions applied in C-AST were not shown to induce significant stress on the backsheet.

**Figure 9:**
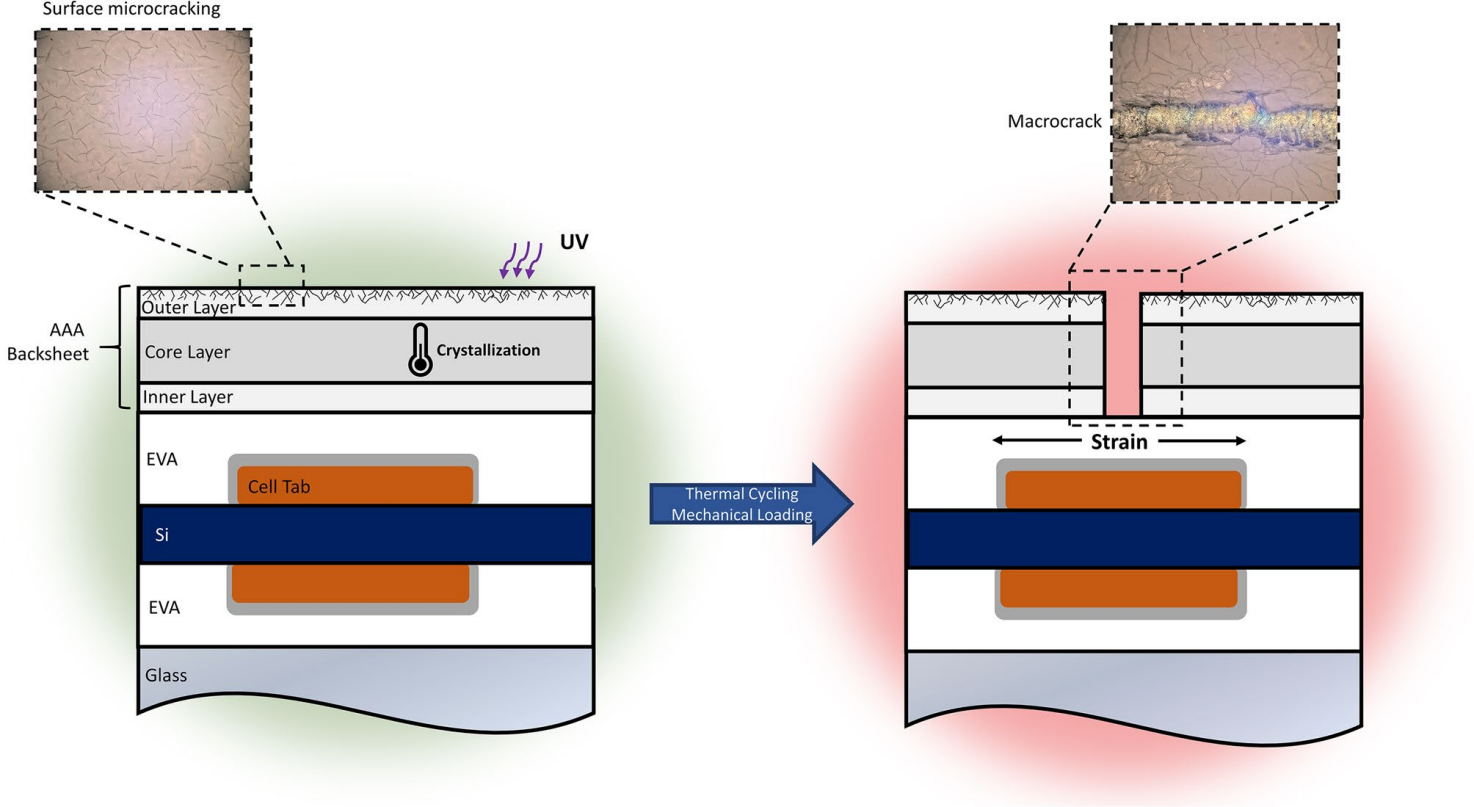
Schematic of the two-step process for Mode A cracking of AAA, demonstrating the required combination of stressors which are applied in C-AST. Graphic rendered using Microsoft PowerPoint (16002.12325.20032.0), https://www.microsoft.com/en-us/microsoft-365/powerpoint.

In addition to the field- and C-AST-aged samples, two samples were subjected to a UV soak and a thermal soak. Both of which had exposures greater than the C-AST samples, but exhibited little to no degradation. This further supports that the degradation of the AAA backsheet does indeed require a combination of stressors as delivered through C-AST. Existing certification protocols and test standards such as IEC 61215 or IEC TS 62788–7-2 do not deliver the required stress combinations to appropriately evaluate the AAA backsheet, which is the reason that AAA failure was not discovered until after field-deployment. Mode A cracking (over cell tabs) requires a combination of UV, temperature and thermo-mechanical loading (as per Fig. 9).

## Conclusions

In this work, we have demonstrated a step towards validation of the combined-accelerated stress testing method by employing materials forensics to identify the degradation mechanisms of the AAA polyamide-based backsheet aged in C-AST and the field. We found that AAA cracking in the field is complex, with multiple enabling degradation mechanisms, but ultimately is a multi-step process requiring chemical degradation of the inner or outer layers, crystallization of the polypropylene and mechanical loading to initiate fracture. Two cracking modes were defined. Mode A, where macrocracks develop over the cell tabs, and Mode B, where macrocracks are initiated from the inner layer and develop in the gaps between the cells. Both modes require a combination of stressors including UV exposure, thermal cycling and elevated humidity to be induced. All of these conditions are applied in C-AST. Additionally, they are applied appropriately and within the right limits such that no unrealistic degradation modes or mechanisms are being induced.

Here, we have outlined a more comprehensive approach to materials development and testing which goes beyond the current certification protocols. This includes more appropriate stress testing using combined stressors which more accurately simulates the natural environment, followed by in-depth materials forensics to identify, and understand potential weaknesses prior to commercialization and deployment. While WAXS is not always a readily available characterization technique, FTIR, DSC and microscopy are more readily available techniques and are deemed crucial to characterizing changes in material properties. Through this approach, more robust materials may be developed, thus minimizing the risk of early, widespread failures and extending lifetimes.

## Supplementary Information

Supplementary Information.
