# Supplementary material for: Towards validation of combined-accelerated stress testing through failure analysis of polyamide-based photovoltaic backsheets
Source: Sci Rep. 2021 Jan 21;11:2019. doi: 10.1038/s41598-021-81381-7 (PMC7820324; doi:10.1038/s41598-021-81381-7)
Supplement: Supplementary file 1 — Supplementary Information. [file 41598_2021_81381_MOESM1_ESM.docx]

**Supplementary Information** **for**

**“Towards Validation of Combined-Accelerated Stress Testing through Failure Analysis of Polyamide-based Photovoltaic backsheets”**

Michael Owen-Bellini^*1^, Stephanie L. Moffitt^2^, Archana Sinha^2^, Ashley M. Maes^3^, Joseph J. Meert^3^, Todd Karin^4^, Chris Takacs^2^, Donald R. Jenket^1^, James Y. Hartley^3^, David C. Miller^1^, Peter Hacke^1^, Laura T. Schelhas^1,2^

^1^National Renewable Energy Laboratory, ^2^SLAC National Accelerator Laboratory, ^3^Sandia National Laboratories, ^4^Lawrence Berkeley National Laboratory

Optical Microscopy

Representative high dynamic range digital images of the specimen surfaces were collected using an optical microscope (VHX-5000, Keyence Corp), equipped with polarizers (OP-72406 and OP-87800, Keyence Corp) to maximize the color contrast of the bright field images.

Wide-Angle X-ray Scattering

WAXS data were collected at the Stanford Synchrotron Radiation Lightsource (SSRL), beamline 11–3 at the SLAC National Accelerator Laboratory. The 2D scattering data were collected using a Rayonex MX225 detector in transmission geometry. The X-ray photon energy used was 12.7 keV (0.9744 Å). Images were calibrated using a LaB6 standard and integrated using GSAS‐II software.

X-ray Transmission Imaging

X-ray imaging was performed at SSRL beamline 2-2 using a monochromatic light (9.6 keV) and a home-built X-ray microscope consisting of a Ce:YAG scintillator imaged with 10X microscope and a visible light CMOS camera.

Fourier-Transform Infrared Spectroscopy

FTIR was performed with a Nicolet Nexus 2400 (Thermo Fisher Scientific, LLC.). Attenuated total reflectance (ATR) spectra were obtained from 4000 to 675 cm^-1^ in 4 cm^-1^ increments using an ART Max-II (PIKE technologies) 50 mm × 10 mm × 5 mm ZnSe crystal attachment. A sampling depth of approximately 2 μm was used. An EverGlo mid-infrared blackbody source (Thermo Fisher Scientific, LLC.) was used as a light source with the crystal pressed against the surface of each test specimen. To account for the variability of measurement and measurement variation between the specimens, the magnitude of the measured spectra was subsequently normalized to the 1465 cm^-1^ peak, and the spectra minima between 600 cm^-1^ and 4000 cm^-1^ were offset relative to zero. To accommodate measurements in the Nicolet Nexus 2400, the specimens were diced into ~5 cm × ~10 cm coupons using an abrasive water-jet saw (Sterling Edge Industrial Cutting, LLC).

Differential Scanning Calorimetry

DSC was completed using a Q200 DSC (TA Instruments, Inc.) with a liquid nitrogen cooling system. Circular samples 0.3 inches in diameter and weighing approximately 10 mg were tested in aluminum pans with lids from TA Instruments. The temperature was ramped at a rate of 5 °C/min from 40 °C to 200 °C and back down to -100 °C. Conditions were selected to monitor the sample through melting and recrystallization thermal transitions.


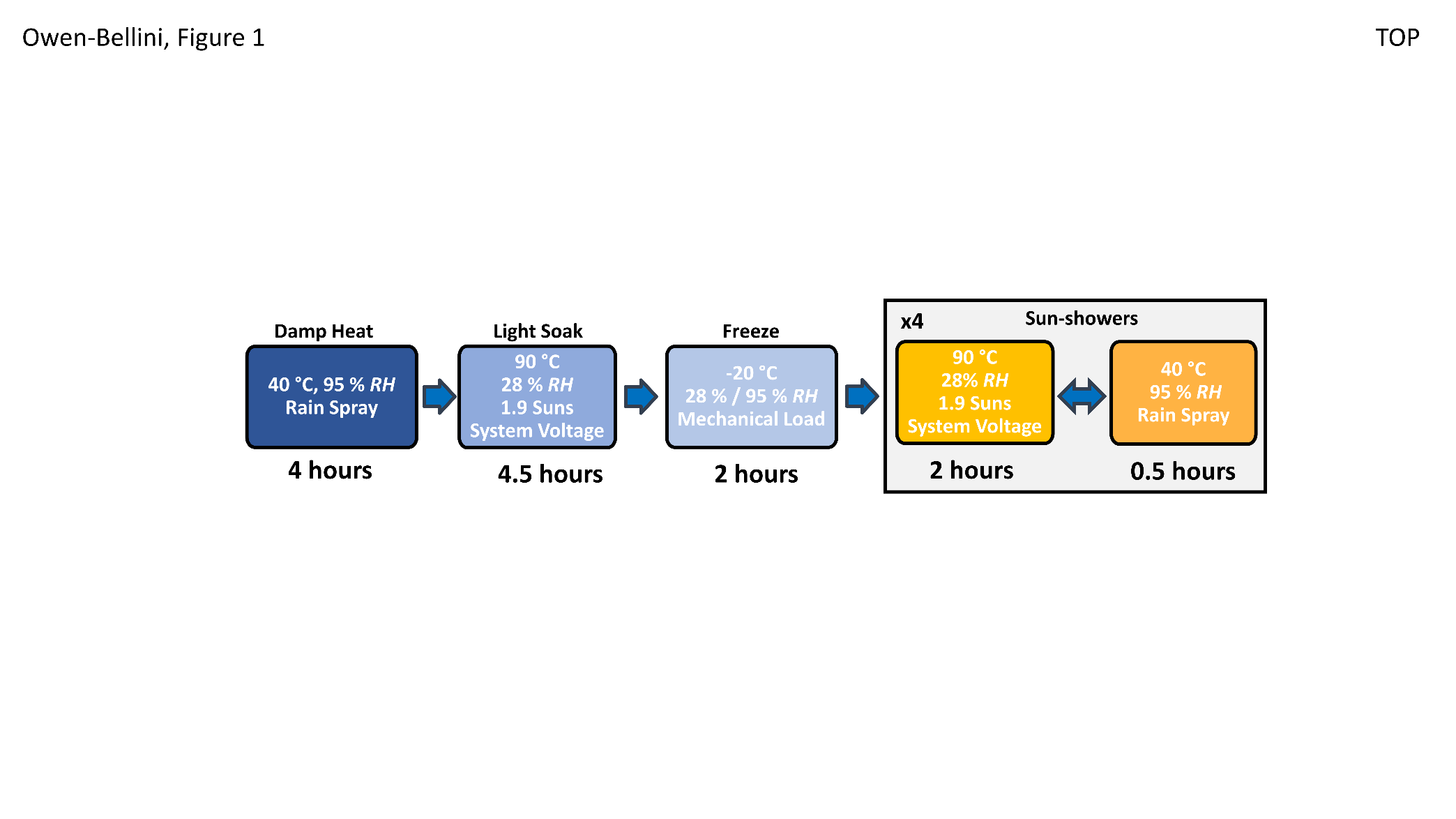


Figure S1: Schematic for the “Tropical” test sequence, where indicated temperatures refer to module temperature. The first 4.5-hour light soak stage has a 30-min 0.8 sun ramp up and ramp down to simulate diurnal cycling. Subsequent 2-hour light soak stages have a 0.8 sun, 20-min ramp up to simulate rain showers. Graphic rendered using Microsoft PowerPoint (16002.12325.20032.0), https://www.microsoft.com/en-us/microsoft-365/powerpoint


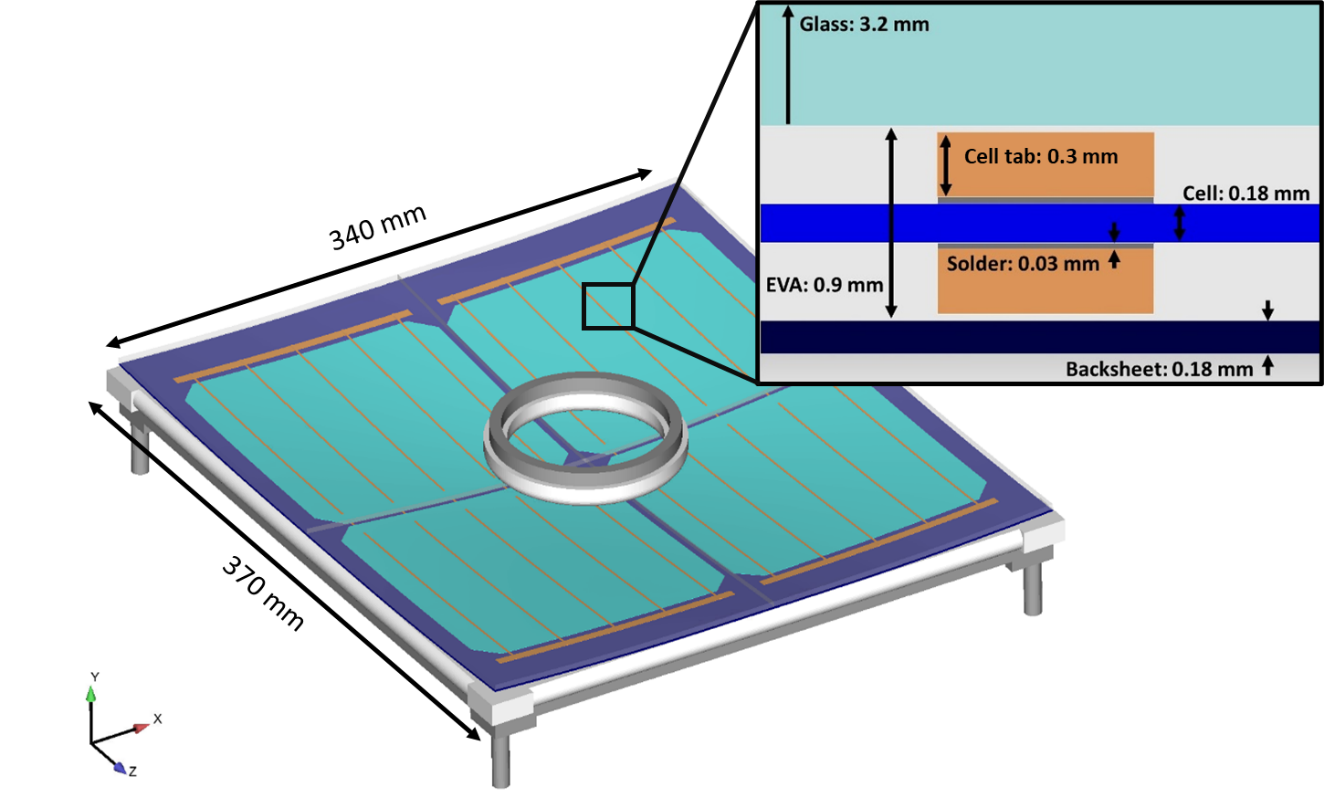


Figure S2: Schematic of the C-AST mini-module finite-element model with inset showing interconnect and layer thickness dimensions. Graphics rendered using Sierra/SolidMechanics 4.56, https://www.sandia.gov/ASC/integrated_codes.html.


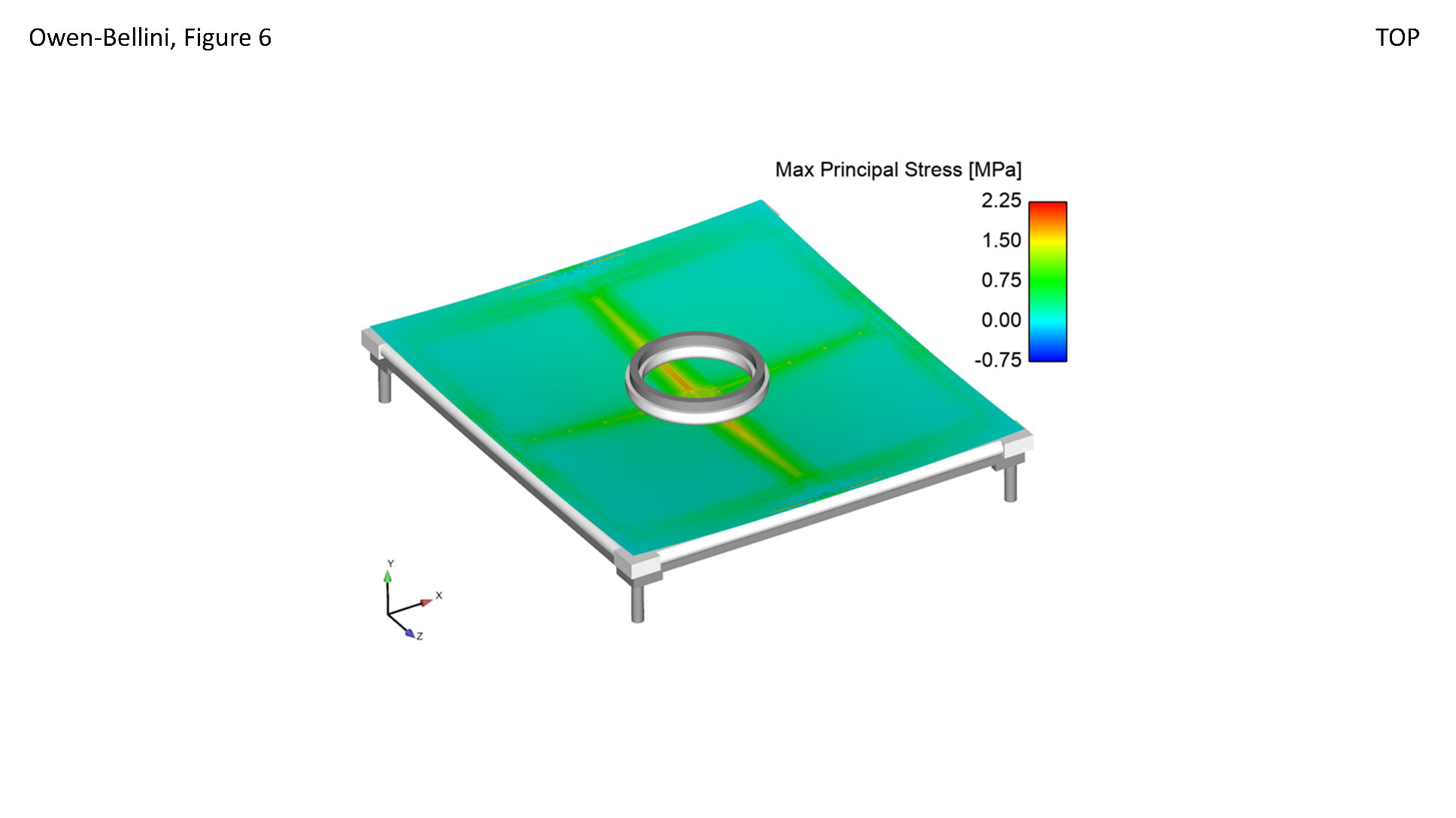


Figure S3: Simulated principal stress on backsheet for C-AST MiMo at 25 °C reference state under mechanical load (1.1 mm deflection at the center), showing stress concentrations in cell gap areas. Displacements scaled by 10x. Graphics rendered using Sierra/SolidMechanics 4.56, https://www.sandia.gov/ASC/integrated_codes.html.

Table S1: Summary of fielded samples, field location and times, and results of visual inspection

| Module ID | Location | Time (yrs.) | Visual Observations |
| --- | --- | --- | --- |
| Tonopah | Tonopah, Arizona, USA | 3 | Microcracking, Chalking |
| Changshu | Changshu, China | 4 | Microcracking, Chalking |
| Rome | Rome, Italy | 5 | Macrocracking (Mode B), Chalking |
| Bergamo | Bergamo, Italy | 6 | Macrocracking (Mode B), Chalking |

Table S2: Summary of indoor test samples, stress test types and times, and results of visual inspection

| Module Id | Exposure | Observations |
| --- | --- | --- |
| Unaged | N/A | N/A |
| C-AST-1 | C-AST 84 days | Chalking |
| C-AST-2 | C-AST 184 days | Macrocracking (Mode A) |
| UV | UV, 45 °C, 63 days | No visual Change |
| T | 90 °C, 83 days | Chalking |
